# Population-based laboratory surveillance for *Giardia *sp. and *Cryptosporidium *sp. infections in a large Canadian health region

**DOI:** 10.1186/1471-2334-5-72

**Published:** 2005-09-16

**Authors:** Kevin B Laupland, Deirdre L Church

**Affiliations:** 1 Departments of Critical Care Medicine, Medicine and Pathology & Laboratory Medicine, and Community Health Sciences, University of Calgary, Calgary, Alberta, Canada; 2 Center for Anti-microbial Resistance, Calgary Health Region, Calgary Laboratory Services and the University of Calgary, Calgary, Canada; 3 c/o Calgary Laboratory Services, 9-3535 Research Rd. N.W., Calgary, Alberta, T2L 2K8, Canada; 4 Departments of Pathology & Laboratory Medicine and Medicine, University of Calgary, Calgary, Alberta, Canada; 5 Calgary Laboratory Services, Calgary, Alberta, Canada

## Abstract

**Background:**

*Giardia lamblia *(*intestinalis*) and *Cryptosporidium parvum *are the two most important intestinal parasites infecting North Americans but there is a paucity of active population-based surveillance data from Canada. This study determined the incidence of and demographic risk factors for developing *Giardia *sp. and *Cryptosporidium *sp. infections in a general Canadian population.

**Methods:**

Population-based laboratory surveillance was conducted among all residents of the Calgary Health Region (CHR; population ≅ 1 million) during May 1, 1999 and April 30, 2002.

**Results:**

*Giardia *sp. infection occurred at a rate of 19.6 per 100,000 populations per year. Although the yearly incidence was stable, a significant seasonal variation was observed with a peak in late summer to early fall. Males were at higher risk for development of this infection as compared to females (21.2 vs. 17.9 per 100,000/yr; relative risk (RR) 1.19; 95% confidence interval (CI), 1.00–1.40, p = 0.047), and there was a significant decrease in risk associated with an increasing age. *Cryptosporidium *sp. infection occurred at an overall rate of 6.0 per 100,000 populations per year although a large outbreak of *Cryptosporidium *sp. infections occurred in the second half of the summer of 2001. During August and September of 2001, the incidence of cryptosporidiosis was 55.1 per 100,000 per year as compared to 3.1 per 100,000 per year for the remainder of the surveillance period (p < 0.0001). Cryptosporidiosis was largely a disease of children with an incidence of 17.8 per 100,000 per year occurring among those aged < 20 years of age compared to 1.25 per 100,000 per year for adults ≥ 20 years of age (RR 14.19; 95% CI, 9.77–21.11; p < 0.0001).

**Conclusion:**

This study provides important information on the occurrence and demographic risk groups for acquisition of giardiasis and cryptosporidiosis in a non-selected Canadian population.

## Background

*Giardia lamblia *(*intestinalis*) and *Cryptosporidium parvum *are the two most important intestinal parasites infecting North Americans [1,2]. Infection with either parasite occurs when cysts are ingested via contaminated hands, food and/or water, or through person to person contact [3,4]. These infections are associated with endemic infection rates in many jurisdictions but they have also caused large outbreaks [5,6]. In Canada, several outbreaks have been reported in recent decades due to *Cryptosporidium parvum *[7,8] and *Giardia lamblia *[9]. Population-based surveillance studies conducted in the United States have demonstrated increasing rates for giardiasis between 1992–97 with the highest national rates being amongst children aged 0–5 years, followed closely by persons aged 31–40 years [1,10-12]. In the United States in 1997, giardiasis cases per 100,000 state population ranged from 0.98 to 42.3 with a national average of 9.5 cases per 100,000 population [1]. Similar true population-based rates have not been determined for cryptosporidiosis in North America. Crude estimates from the United States based on monitoring water contamination show an expected *Cryptosporidium *sp. infection rate of between 1–400 cases per 100,000 population [13].

However, because water-borne transmission is one of the major routes of acquiring infection, it must also be recognized that infection rates for either enteric parasite may vary by geographic location. One study in southern Ontario demonstrated an association between giardiasis and rural areas and this has been corroborated by subsequent GIS spatial scan statistics investigation of clusters of giardiasis in this area [14]. Although a previously study had shown significant associations of giardiasis rates with manure application on agricultural land and livestock density this was not born out using spatial statistics scanning methods [15].

There is a lack of active population-based data on the distribution and determinants of *Giardia *sp. and *Cryptosporidium *sp. infections in a Canadian population. The objective of this study was to determine the incidence of and demographic risk factors for acquiring these infections among residents of the Calgary Health Region. Such data is important to establish the burden of disease and assess risk factors for acquiring these infections in a defined geographic locale.

## Methods

### Patients

The Calgary Health Region (CHR) is a well defined, fully integrated, publicly funded health system that provides virtually all medical and surgical care to the residents of the cities of Calgary and Airdrie and several nearby small towns, villages, and hamlets (2001 population 958,610) [16]. All residents of the CHR who had a positive stool specimen for *Giardia *sp. or *Cryptosporidium *sp. during the period from May 1, 1999 and April 30, 2002 were included in this study.

### Study design

A laboratory-based surveillance cohort design was utilized since all stool samples for parasitological testing are routinely submitted to a single regional microbiology laboratory [i.e., Calgary Laboratory Services (CLS)] [17]. CHR physicians order stool tests based on the patient's presenting symptoms typically of a diarrhoeal illness and the presence of other risk factors (i.e., recent travel, exposure to contaminated food or water, immune status etc.). Because there is universal coverage for healthcare services including laboratory tests in Canada, lack of health insurance is not a barrier to stool parasitological testing. All stool samples submitted from community-based or hospital collection sites in the CHR during the study period were identified by use of the Cerner PathNet Classic version 306 (Kansas City, MO) database at Calgary Laboratory Services (CLS) [18]. Patients were deemed to have giardiasis or cryptosporidiosis if an approved diagnostic test was positive for one or both of these infections according to the laboratory procedures outlined below. Once these patients were identified basic demographic information on age and gender was obtained at CLS and data were entered manually into an Excel spreadsheet (Microsoft Corp.).

### Laboratory procedures

All stool parasitological tests were performed by Calgary Laboratory Services (CLS) a large integrated publicly funded medical laboratory company that provides microbiology services from hospitalized and ambulatory patients 24 hours a day, 7 days a week through a centralized facility located in the community. Physicians can order either a *Giardia*/*Cryptosporidium *screen or a full stool ova and parasite (O & P) examination on the CLS requisition. Stool parasitological testing proceeded as outlined below depending on the physician's test request. Only a G/C EIA screen was done unless a clinical history was provided on the requisition. All stool specimens that had a stool O & P procedure were initially screened by the G/C EIA procedure.

#### a) Giardia/Cryptosporidium assay

A rapid commercially available immunoassay is initially performed when a G/C screen is ordered. A stained slide was also read from all stool samples submitted from children ≤ 14 years of age) in order to screen for the presence of other parasites including *Dientamoeba fragilis*. *Giardia *(GSA 65) and *Cryptosporidium *(CSA) are specific antigens produced by the parasites as they multiply within the host intestinal tract. The ProSpecT^® ^*Giardia*/*Cryptosporidium *Microplate (Remel Inc., Lenexa, KS, USA) assay (G/C EIA) is a solid phase immunoassay for the simultaneous detection of both antigens (GSA 65 and CSA). The G/C EIA was performed according to the manufacturer's instructions on unconcentrated formalin-fixed specimens. G/C EIA tests were considered positive if the optical density was 0.050 ≥ at 450 nm. The Meriflor^® ^G/C direct fluorescent antibody (DFA) test (Meridian Bioscience Inc., Cincinnati, OH) was used to confirm the presence of *Giardia *and/or *Cryptosporidium *infection. One drop (~10 μl) of the concentrated sediment was thinly spread onto each well of the treated slide and fluorescence light microscopy with a 20X objective was used to read the entire well.

#### b) Stool ova & parasite (stool O & P) method

Stool O & P testing (i.e. stained slide and concentrate) were only done on samples where the physician had ordered the test and provided one or more clinical indications for the request as follows: 1) recent travel outside North America or Travel Clinic patient, 2) recent immigration, 3) immune-compromised or specific HIV/AIDS, cancer, transplantation clinic locations, 4) request for worms, tapeworms or *Ascaris *sp., 5) bloody or bloody stool specimen, 6) Gastroenterologist's patient, and 7) other (Microbiologist-on-call is consulted). Repeated standard wash and centrifugation procedures are used to prepare stool sediment that is stained with Iron Hematoxylin/Kinyoun stain. The entire 20 × 50 mm cover-slipped stained smear is scanned with the 10X objective to look for larger parasitic elements (i.e. ova). The stained slide is then examined for 10 min. per slide using the 50X oil immersion lens and confirmation of any parasitic elements is done under the 100X oil immersion lens. A fecal concentrate is also prepared using a standard ethyl acetate concentration method after preparation of the stained smears. A different technologist reads the concentrate of the same specimen using both the 10X and 40X dry objectives.

### Analysis

Analyses were performed using Stata version 8.0 (Stata Corp., College Station, TX). Differences in proportions in categorical data were compared using Fisher's exact test. Medians with interquartile ranges (IQR) were used to report non-normally distributed continuous variables and were compared using the Wilcoxon Rank-sum test. Incidence rates were calculated using denominator data from the Alberta Health Registry [19]. Only the first sample positive in a given study year was included in the analysis. Category specific risks were calculated and reported as relative risks (RR) with 95% confidence intervals (CI) as previously described [20]. Patients were classified as CHR residents if they had Alberta Health Care numbers and samples were collected at a CHR site or if they were inpatients in a CHR hospital.

## Results

### Giardiasis

**Figure 1:**
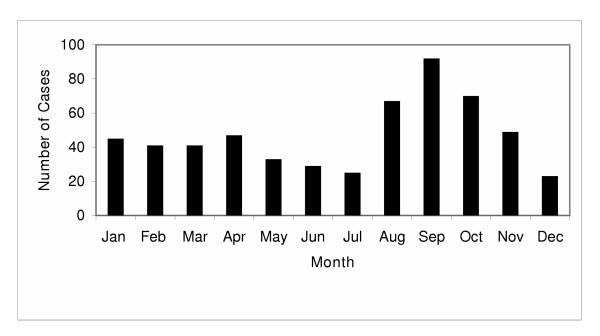
Seasonal distribution of *Giardia *sp. infections in the Calgary Health Region.

A total of 562 episodes of *Giardia *sp. infection were identified among 552 patients for an overall incidence of 19.6 per 100,000 populations per year. There was no difference in the yearly incidence of *Giardia *sp. infection during the three years of the study (p > 0.2). A significant seasonal variation was observed with a peak in late summer to early fall (Figure 1). The incidence rate in the second quarter of the study (August to October; 31.9 per 100,000/year) was two times higher (relative risk (RR), 2.06; 95 % confidence interval (CI), 1.74–2.45) than in the other three quarters (November to July, 15.5 per 100,000/year; p < 0.0001).

**Figure 2:**
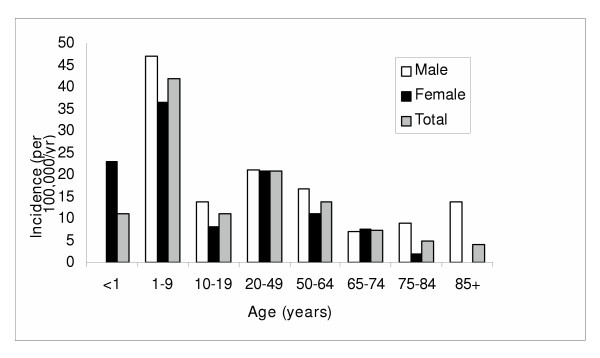
Age and gender specific incidence rates of *Giardia *sp. infection in the Calgary Health Region.

A significant association between age and gender and development of giardiasis was observed (Figure 2). Overall males were at slightly higher risk for development of this infection as compared to females (21.2 vs. 17.9 per 100,000/yr; RR 1.19; 95% CI, 1.00–1.40, p = 0.047). There was a significant decrease in risk associated with a increasing age with children and adolescents less than 20 years of age at highest risk (24.6 per 100,000 per year) followed by adults aged 20 to 64 (19.4 per 100,000 per year), and senior adults aged 65 years and older (6.2 per 100,000 per year; p < 0.01 for each pairwise comparison).

### Cryptosporidiosis

**Figure 3:**
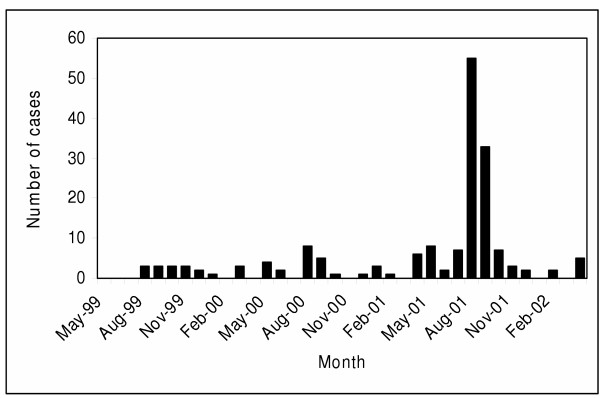
Occurrence of infections with *Cryptosporidium *sp. in the Calgary Health Region.

A total of 173 patients were diagnosed with *Cryptosporidium *sp. infection for an overall incidence of 6.0 per 100,000 populations per year. No patients had repeat episodes of this infection. There was a dramatic difference in the temporal occurrence of *Cryptosporidium *sp. infection during the three years of the study with a large outbreak occurring in the second half of the summer of 2001 (Figure 3). During August and September of 2001, 88 patients were diagnosed with cryptosporidiosis for an incidence of 55.1 per 100,000 per year as compared to 3.1 per 100,000 per year for the remainder of the surveillance period (p < 0.0001).

**Figure 4:**
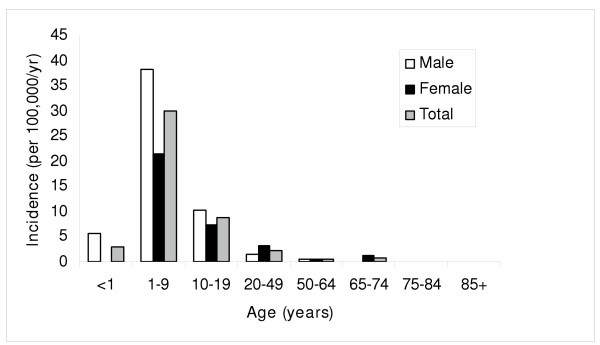
Age and gender specific incidence rates of *Cryptosporidium *sp. infection in the Calgary Health Region.

In contrast to giardiasis where adults remained at notably increased risk, cryptosporidiosis was primarily a disease of children and adolescents (Figure 4). A total of 137 (79%) cases were in individuals < 20 years of age for an incidence of 17.8 per 100,000 per year as compared to 1.25 per 100,000 per year for adults ≥ 20 years of age (RR 14.19; 95% CI, 9.77–21.11; p < 0.0001). However, these findings are influenced by the fact that significant differences in age existed among outbreak related (August and September 2001) as compared to non-outbreak (other study surveillance periods) related patients with *Cryptosporidium *sp. infections. The median (IQR) age of outbreak related patients were significantly lower than that of non-outbreak patients (7.5 years; IQR; 3.5–11 years vs. 10 years; IQR, 4–23 years, p = 0.02). Ninety-two (81/88) percent of the outbreak patients were < 20 years of age as compared to 66% (56/85) of non-outbreak patients (p < 0.0001).

Males were at slightly higher risk overall for development of cryptosporidiosis as compared to females (6.9 vs. 5.1 per 100,000/yr; RR 1.35; 95% CI, 0.99–1.84, p = 0.053) which was similar to the trend found for giardiasis. No significant association was observed between gender and the presence of the outbreak.

## Discussion

This is the first study that has investigated the occurrence and demographic risk factors for acquiring *Giardia *sp. and *Cryptosporidium *sp. infections using a population-based methodology in Canada. Population-based methodologies are widely viewed as optimal designs for establishing the burden of disease because selection bias is minimized since all patients in a defined geographic area are included in surveillance [21-23]. The American Centers for Disease Control (CDC) that is recognized as a leader in infectious disease epidemiology utilizes a very similar methodology for their Active Bacterial Core Surveillance program .

If adequate denominator data is available then incidence rates may be calculated from population-based datasets. The rates we determined in this study should be viewed as the minimum incidence because multiple stool sampling is not a standard practice in our region, and many patients may have had these diseases without having a stool sample submitted for parasitological testing. In addition, certain groups that seek medical attention less frequently may be underrepresented using a population-based laboratory surveillance approach (i.e., shut-in elderly, intravenous drug users, those with mental illness etc.), but these limitations would also be a reality of other approaches. However, even considering these limitations, *Giardia *and *Cryptosporidium *result in a significant disease burden in Calgary. We found that infections with *Giardia *sp. and/or *Cryptosporidium *sp. were common in our region.

Epidemiological analysis of regional stool parasitological testing data over a two-year period demonstrated that these two parasites were the most commonly identified pathogenic enteric protozoa in the Calgary Health Region, which is similar to the rates previously reported in the United States [1,15,24]. Population-based studies in the United States have indicated rates of *Giardia *sp. infections ranging from 0.98 to 42.3 cases per 100,000 per year with an average of 9.5 cases per 100,000 per year [1]. However, true population based rates for *Cryptosporidum *sp. based on laboratory surveillance data have been difficult to determine in the United States because most diagnostic laboratories do not routinely perform stool parasitological procedures that would allow detection of this parasite. A recent survey indicates that most laboratories only analyze stool parasitological samples for the presence of *Cryptosporidium sp*., *Cyclspora *sp. and *Microsporidium *sp. on specific request of the physician [25]. Based on laboratory surveillance data from clinical microbiology laboratories, there were a total of 443 cases of cryptosporidiosis reported to the CDC in the United States in 2003 [26] which is much lower than that expected from population-based estimates of infection rates. Estimates for the *Cryptosporidium *sp. infection rates for the general population (i.e., 1 – 400 cases per 100,000) in the United States have been based on US-wide water monitoring data [27]. The population-based rate of giardiasis and cryptosporidiosis cases in Calgary is similar to the national average rate per 100,000 reported from the United States. However, the rate of *Cryptosporidium *sp. infections found in our region is well above the proposed acceptable annual risk level of 1 case of infection per 10,000 [13]. Substantially more *Cryptosporidium *sp. infections were found in our region during the study period. To our knowledge studies conducted to date from Canada have also not been adequately designed to report incidence rates to allow comparison. The results of our study demonstrate that *Giardia *sp. and *Cryptosporidium *sp. infections are common with the former occurring at a rate of more than 3 times more frequently.

We observed a number of similar and contrasting features among infections due to *Giardia *and *Cryptosporidium *sp. *Giardia *sp. infections occurred more commonly in late summer in our region and may be related to water exposure and the increased use of backcountry recreational areas. On the other hand, there was no apparent yearly seasonal occurrence of sporadic (i.e. non-outbreak related) *Cryptosporidium *sp. infections in our region (Figure 3). Although few studies of the seasonal incidence of intestinal parasites have been done, prior reports from specific geographic locations in the United States suggest that *Cryptosporidium *sp. infection is most prevalent in the spring, while no seasonality was found in *Giardia *sp. infections [28]. Infection with either parasite occurs when cysts are ingested via contaminated hands, food, and/or water or through person-to-person contact. Individual cases as well as outbreaks have therefore been associated with improper food handling, exposure to contaminated water (i.e. municipal sources including swimming pools, surface and groundwater including those found in beaver ponds and springs), travel to less developed countries or close contact with a case (i.e. families, day care centers) [12,29-33]. Specific risk factors that have recently been associated with sporadic cryptosporidiosis among immunocompetent persons in the United States include international travel, contact with cattle, contact with persons >2 to 11 years of age with diarrhea and freshwater swimming [34]. Temporal investigation of fecal shedding of *Cryptosporidium parvum *oocysts in cow-calf herds has also shown that most fecal shedding of this parasites by infected cattle is limited to calves 1 to 4 months old so that the risk of parasitic watershed contamination is greatest in the spring during calving season [35]. Enteric parasitic infection with either *Giardia *sp. or *Cryptosporidium *sp. may also be transmitted through sexual contact and immunocompromised persons (i.e. acquired immunodeficiency syndrome) are particularly at risk of developing severe persistent infection [36]. Differences in the seasonality of intestinal parasite prevalence between disparate geographic locations may therefore be dependent on a variety of factors including the composition of the population and their coming into contact with various contaminated water sources (i.e. urban versus rural areas).

Another contrasting feature among these infections was that the age distribution was different. *Giardia *sp. infections, although most commonly infecting children, occurred across a broad age range (Figure 2). In contrast, *Cryptosporidum *sp. infections occurred primarily in young children. The main commonality amongst these two infections was that they both tended to more commonly affect males as compared to females [11,37]. Although this study did not investigate the risk factors associated with disease acquisition in various age groups, the demographics of infection is similar to prior studies [11,13,34,37]. Young children tend to have a higher rate of infection with *Giardia *sp. and/or *Cryptosporidium *sp. because of their exposure to contaminated municipal and rural water sources such as well water and swimming pools and contact with cases in communal living situations including day care centers. Epidemiological investigation into the risk factors for enteric infection due to one or both of these parasites is the focus of future studies in our region.

Our observation that an outbreak of *Cryptosporidium *sp. occurred in the third year of the study demonstrates the importance of their detection by conducting population-based studies over extended periods (years). We have previously raised the theoretical potential for bias in population-based studies designed to assess the burden and risk factors for disease if they are conducted in the setting of an outbreak [38]. If this study had only been conducted over a one-year period in 2001 we would have made erroneous inferences on the occurrence and demographic risk factors for endemic disease as evidenced by the dramatically different rate in that year and that outbreak patients were younger. In our opinion it is important that "baseline" or endemic disease be differentiated from outbreak cases in the description of the epidemiology of an infectious disease. We urge authors of other studies to plot cases over time prior to detect outbreaks prior to description.

Although our study is novel in Canada and provides important information there are some important limitations that merit discussion. First, this was a laboratory based study and as a result we did not have detailed information on patient clinical variables, treatments, and outcome. Such information may be of interest and use to clinicians. Second, we did not have reliable other data on exposures such as food and water sources, travel, and family exposures. These variables are clearly important in these diseases and have been the focus of other important investigations in Canadian populations [2,12,29,32]. Finally, we did not have actual addresses for all patients and used a definition of CHR residency status that may have resulted in at least some non-CHR residents being classified as residents in our study. As a result we likely overestimated the incidence of these infections and may have made biased assessments of demographic risk factors to at least some degree [21].

## Conclusion

In conclusion, we present the results of active population-based surveillance for *Giardia *sp. and *Cryptosporidium *sp. in a large Canadian region. Giardiasis was endemic, and younger individuals and males were at increased risk. Overall, cryptosporidiosis was much less common than giardiasis, although a large outbreak of cryptosporidiosis occurred during surveillance. These data provide important information on the occurrence and determinants of the two most important intestinal parasitoses in Canada.

## Competing interests

The author(s) declare that they have no competing interests.

## Authors' contributions

DLC performed the laboratory surveillance study at Calgary Laboratory Services and KBL performed the data analysis. Both authors wrote the manuscript.

## Pre-publication history

The pre-publication history for this paper can be accessed here:



## References

[B1] Furness BW, Beach MJ, Roberts JM (2000). Giardiasis surveillance – United States, 1992–1997. MMWR CDC Surveill Summ.

[B2] Croll NA, Gyorkos TW (1979). Parasitic disease in humans: the extent in Canada. Can Med Assoc J.

[B3] Odoi A, Martin SW, Michel P, Holt J, Middleton D, Wilson J (2004). Determinants of the geographical distribution of endemic giardiasis in Ontario, Canada: a spatial modelling approach. Epidemiol Infect.

[B4] Welch TP (2000). Risk of giardiasis from consumption of wilderness water in North America: a systematic review of epidemiologic data. Int J Infect Dis.

[B5] Yoder JS, Blackburn BG, Craun GF, Hill V, Levy DA, Chen N, Lee SH, Calderon RL, Beach MJ (2004). Surveillance for waterborne-disease outbreaks associated with recreational water – United States, 2001–2002. MMWR Surveill Summ.

[B6] Hayes EB, Matte TD, O'Brien TR, McKinley TW, Logsdon GS, Rose JB, Ungar BL, Word DM, Pinsky PF, Cummings ML (1989). Large community outbreak of cryptosporidiosis due to contamination of a filtered public water supply. N Engl J Med.

[B7] Louie K, Gustafson L, Fyfe M, Gill I, MacDougall L, Tom L, Wong Q, Isaac-Renton J (2004). An outbreak of Cryptosporidium parvum in a Surrey pool with detection in pool water sampling. Can Commun Dis Rep.

[B8] Stirling R, Aramini J, Ellis A, Lim G, Meyers R, Fleury M, Werler D (2001). Waterborne cryptosporidiosis outbreak, North Battleford, Saskatchewan, spring 2001. Can Commun Dis Rep.

[B9] Wallis PM, Matson D, Jones M, Jamieson J (2001). Application of monitoring data for Giardia and Cryptosporidium to boil water advisories. Risk Anal.

[B10] Dietz VJ, Roberts JM (2000). National surveillance for infection with Cryptosporidium parvum, 1995–1998: what have we learned?. Public Health Rep.

[B11] Addiss DG, Davis JP, Roberts JM, Mast EE (1992). Epidemiology of giardiasis in Wisconsin: increasing incidence of reported cases and unexplained seasonal trends. Am J Trop Med Hyg.

[B12] Isaac-Renton JL, Philion JJ (1992). Factors associated with acquiring giardiasis in British Columbia residents. Can J Public Health.

[B13] Makri A, Modarres R, Parkin R (2004). Cryptosporidiosis susceptibility and risk: a case study. Risk Anal.

[B14] Odoi A, Martin SW, Michel P, Middleton D, Holt J, Wilson J (2004). Investigation of clusters of giardiasis using GIS and a spatial scan statistic. Int J Health Geogr.

[B15] Odoi A, Martin SW, Michel P, Holt J, Middleton D, Wilson J (2003). Geographical and temporal distribution of human giardiasis in Ontario, Canada. Int J Health Geogr.

[B16] Calgary Health Region Website. http://www.ozcan.ca/chr_map/.

[B17] Pitout JD, Hanson ND, Church DL, Laupland KB (2004). Population-based laboratory surveillance for Escherichia coli-producing extended-spectrum beta-lactamases: importance of community isolates with blaCTX-M genes. Clin Infect Dis.

[B18] Church D, Hall P (1999). Centralization of a regional clinical microbiology service: The Calgary experience. Can J Infect Dis.

[B19] Calgary Health Region Website. http://www.calgaryhealthregion.ca/hocr/influ/demo/popage.htm.

[B20] Laupland KB, Davies HD, Low DE, Schwartz B, Green K, McGeer A (2000). Invasive group A streptococcal disease in children and association with varicella-zoster virus infection. Ontario Group A Streptococcal Study Group. Pediatrics.

[B21] Laupland KB (2004). Population-based epidemiology of intensive care: critical importance of ascertainment of residency status. Crit Care.

[B22] Davies HD, McGeer A, Schwartz B, Green K, Cann D, Simor AE (1996). Invasive group A streptococcal infections in Ontario, Canada. Ontario Group A Streptococcal Study Group. N Engl J Med.

[B23] Laupland KB, Gregson DB, Zygun DA, Doig CJ, Mortis G, Church DL (2004). Severe bloodstream infections: a population-based assessment. Crit Care Med.

[B24] Crompton DW, Savioli L (1993). Intestinal parasitic infections and urbanization. Bull World Health Organ.

[B25] Jones JL, Lopez A, Wahlquist SP, Nadle J, Wilson M (2004). Survey of clinical laboratory practices for parasitic diseases. Clin Infect Dis.

[B26] Centers for Disease Control (CDC) (2004). Preliminary FoodNet data on the incidence of infection with pathogens transmitted commonly through food – Selected sites, United States, 2003. MMWR.

[B27] U.S. Environmental Protection Agency. Office of Water (2001). Human Health Criteria Document for Cryptosporidium (Publication No. EPA-822-K-94-001).

[B28] Amin OM (2002). Seasonal prevalence of intestinal parasites in the United States during 2000. Am J Trop Med Hyg.

[B29] Isaac-Renton J, Blatherwick J, Bowie WR, Fyfe M, Khan M, Li A, King A, McLean N, Medd L, Moorehead W, Ong CS, Robertson W (1999). Epidemic and endemic seroprevalence of antibodies to Cryptosporidium and Giardia in residents of three communities with different drinking water supplies. Am J Trop Med Hyg.

[B30] Gradus M (2000). Cryptosporidium and public health: From watershed to water glass. Clin Microbiol News.

[B31] Okhuysen PC (2001). Traveler's diarrhea due to intestinal protozoa. Clin Infect Dis.

[B32] Keystone JS, Krajden S, Warren MR (1978). Person-to-person transmission of Giardia lamblia in day-care nurseries. Can Med Assoc J.

[B33] Thielman NM, Guerrant RL (1998). Persistent diarrhea in the returned traveler. Infect Dis Clin North Am.

[B34] Roy SL, DeLong SM, Stenzel SA, Shiferaw B, Roberts JM, Khalakdina A, Marcus R, Segler SD, Shah DD, Thomas S, Vugia DJ, Zansky SM, Dietz V, Beach MJ (2004). Risk factors for sporadic cryptosporidiosis among immunocompetent persons in the United States from 1999 to 2001. J Clin Microbiol.

[B35] Atwill ER, Johnson E, Klingborg DJ, Verserat GM, Markegard G, Jensen WA, Pratt DW, Delmas RE, George HA, Forero LC, Philips RL, Barry SJ, McDougald NK, Gildsleeve RR, Frost WE (1999). Age, geographic, and temporal distribution of fecal shedding of Cryptosporidium parvum oocysts in cow-calf herds. Am J Vet Res.

[B36] Griffiths JK (1998). Treatment for AIDS-associated cryptosporidiosis. J Infect Dis.

[B37] Clavel A, Olivares JL, Fleta J, Castillo J, Varea M, Ramos FJ, Arnal AC, Quilez J (1996). Seasonality of cryptosporidiosis in children. Eur J Clin Microbiol Infect Dis.

